# Chronic Treatment with Fluoxetine Induces Sex-Dependent Analgesic Effects and Modulates HDAC2 and mGlu2 Expression in Female Mice

**DOI:** 10.3389/fphar.2017.00743

**Published:** 2017-10-20

**Authors:** Magda Zammataro, Sara Merlo, Massimo Barresi, Carmela Parenti, Huijuan Hu, Maria A. Sortino, Santina Chiechio

**Affiliations:** ^1^Department of Drug Sciences, University of Catania, Catania, Italy; ^2^Department of Biomedical and Biotechnological Sciences, University of Catania, Catania, Italy; ^3^Institut des Maladie Neurodégénératives, Bordeaux, France; ^4^Department of Pharmacology and Physiology, Drexel University College of Medicine, Philadelphia, PA, United States

**Keywords:** pain, fluoxetine, sex differences, metabotropic glutamate 2 receptor, HDAC2

## Abstract

Gender and sex differences in pain recognition and drug responses have been reported in clinical trials and experimental models of pain. Among antidepressants, contradictory results have been observed in patients treated with selective serotonin reuptake inhibitors (SSRIs). This study evaluated sex differences in response to the SSRI fluoxetine after chronic administration in the mouse formalin test. Adult male and female CD1 mice were intraperitoneally injected with fluoxetine (10 mg/kg) for 21 days and subjected to pain assessment. Fluoxetine treatment reduced the second phase of the formalin test only in female mice without producing behavioral changes in males. We also observed that fluoxetine was able to specifically increase the expression of metabotropic glutamate receptor type-2 (mGlu2) in females. Also a reduced expression of the epigenetic modifying enzyme, histone deacetylase 2 (HDAC2), in dorsal root ganglia (DRG) and dorsal horn (DH) together with an increase histone 3 acetylation (H3) level was observed in females but not in males. With this study we provide evidence that fluoxetine induces sex specific changes in HDAC2 and mGlu2 expression in the DH of the spinal cord and in DRGs and suggests a molecular explanation for the analgesic effects in female mice.

## Introduction

In recent years gender and sex differences in pain recognition and drug responses have been extensively investigated both in clinical trials and experimental models of pain. Despite a large number of epidemiological studies clearly reveal that women report pain more frequently than men (Unruh, 1996; Berkley, 1997; Riley et al., 1998; Fillingim et al., 2009), results obtained in rodents are not always consistent and often depend on the type of pain and treatment protocol (Craft, 2003; Mogil et al., 2003). More recently, it has been demonstrated that the immune system differently mediates pain hypersensitivity in male and female rodents (Sorge et al., 2015) thus emphasizing the need to elucidate mechanisms underlying sex differences in pain responses with the aim to support the development of gender-specific drug therapies.

Different classes of antidepressant drugs are clinically effective in various forms of chronic pain including arthritis, diabetic neuropathy, post-herpetic neuralgia, migraine, fibromyalgia, low back pain (Gilron et al., 2013). Antidepressant drugs act by blocking the norepinephine and/or serotonin transporter on presynaptic site enhancing the descending inhibitory control on pain (Millan, 2002; Berton and Nestler, 2006; Mika et al., 2013). Nevertheless, many other mechanisms can account for their analgesic activity involving sodium and calcium channels or a number of receptors including GABAB, opioid, and adenosine receptors (Dharmshaktu et al., 2012). In this regard the activity as sodium and calcium blockers as well as the ability to decrease prostaglandin E2 production elicited by several antidepressants including fluoxetine, has been proposed as an important mechanism to explain the efficacy of antidepressants as analgesics (Dharmshaktu et al., 2012).

In clinical practice, it is generally accepted that the tricyclic antidepressant (TCA), amitriptyline, and the serotonin/noreprinephine reuptake inhibitor (SNRI), duloxetine, are effective in persistent pain (Attal et al., 2006; Finnerup et al., 2015). However, there is less agreement about the efficacy of antidepressants belonging to the selective serotonin reuptake inhibitor (SSRI) class (Sindrup et al., 2005; Saarto and Wiffen, 2007; Finnerup et al., 2010). Only few studies have demonstrated some efficacy of SSRIs such as paroxetine and citalopram but not fluoxetine in painful diabetic neuropathy (Sindrup et al., 1990, 1992; Max et al., 1992). On the other hand, in a number of trials that included almost exclusively women, the SSRI fluoxetine, has been shown to be effective in chronic tension-type headache (Walker et al., 1998) and in the treatment of fibromyalgia (Cantini et al., 1994; Wolfe et al., 1994; Goldenberg et al., 1996; Arnold et al., 2002), a musculoskeletal condition characterized by chronic widespread pain and muscle tenderness with a high prevalence on women compared to men (Wolfe et al., 1995).

In rodents the analgesic activity of antidepressant drugs has been evaluated both in models of acute and persistent pain. Most of the studies have been performed with high dose of antidepressants acutely injected (Bomholt et al., 2005; Obata et al., 2005). We have previously shown that fluoxetine is also effective in the formalin test after a single intraperitoneal injection and that the analgesic activity is lost in *Lmx1b* conditional knock-out mice (*Lmx1b^f/f/p^*), which lack 5-HT neurons in the central nervous system (Zhao et al., 2007). However, protocols of acute treatment do not reproduce the clinical use of these drugs.

Based on these pieces of evidence, we sought to investigate whether a long-term administration with fluoxetine might have sex-specific effects in pain behavior in mice. Moreover, antidepressant treatment may induce direct epigenetic effects and fluoxetine itself is known to modulate the expression of epigenetic modifying enzymes (Perisic et al., 2010; Menke et al., 2012; Zimmermann et al., 2012; Menke and Binder, 2014; Erburu et al., 2015; Vierci et al., 2016).

Epigenetic modifications, including DNA methylation and changes of chromatin structure and function, are the most common mechanisms that control gene expression and, eventually, influence protein expression levels. DNA methylation involves direct chemical modification to the DNA, through the addition of a methyl group at the 5′ position of cytosines in CpG dinucleotides, and usually mediate repression of gene transcription (Newell-Price et al., 2000). On the other hand, a number of post-translational modifications of chromatin structure, including acetylation, methylation, phosphorylation may either increase and repress gene expression (Borrelli et al., 2008). In particular, acetylation is regulated by two classes of enzymes, histone acetyltransferases (HATs) and deacetylases (HDACs) that, respectively, promote acetylation and deacetylation of lysine tails in histone proteins (Mai et al., 2005).

We have first demonstrated that HDAC inhibitors produce analgesic effects via the upregulation of metabotropic glutamate receptor type-2 (mGlu2), an inhibitory receptor whose activation mediates analgesic effects in different pain models (Chiechio et al., 2009). Different HDAC proteins have been identified and subdivided in four classes (I, II, III, and IV) according to their structure, cellular localization and function (Sharma et al., 2013). Class I includes HDAC1, HDAC2, HDAC3, and HDAC8. Class II consists of six HDAC proteins that include HDAC4, HDAC5, HDAC6 HDAC7, HDAC9 and HDAC10, while HDAC11 belongs to class IV. Differently to the other HDAC proteins, class III HDACs operate by a NAD^+^-dependent mechanism and are referred to as sirtuins (SIRT1-7) (Grozinger and Schreiber, 2002). However, among HADACs, the HDAC2 is the only isoform that has been shown to regulate the expression of mGlu2 receptors (Kurita et al., 2012) and fluoxetine itself is known to modulate the expression of HDAC2 (Faillace et al., 2015).

With this in mind, we tested whether a chronic treatment with fluoxetine induces sex specific analgesic effects in the mouse formalin test. We also and evaluated HDAC2 and mGlu2 receptor expression in DRG and dorsal horn by western blot and immunohistochemistry analysis.

## Results

### Chronic Treatment with Fluoxetine Reduces the Second Phase of the Formalin Test in Female Mice

**FIGURE 1:**
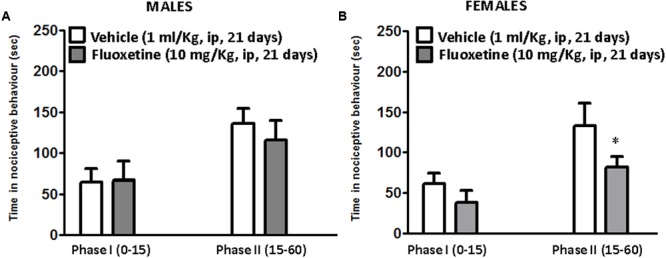
Effects of chronic administration with fluoxetine in the formalin test in male and female CD1 mice. Chronic administration with fluoxetine (10 mg/Kg, i.p. for 21 days) fails to induce analgesia in male mice **(A)** but reduces the second phase of the formalin test in female CD1 mice **(B)**. Data are means ± SEM of 9 to 12 mice per sex/group. ^∗^*p* < 0.05 versus the respective vehicle group (two tailed Student’s *t*-test).

Fluoxetine (10 mg/Kg) was intraperitoneally injected in adult male and female littermates for 21 days and pain behavior was evaluated in the mouse formalin test. The intraplantar (i.pl.) injection of a dilute solution of formalin (10 μl, 5%) induces a biphasic response, namely phase I and phase II, characterized by the flinching, lifting, and licking of the injected paw. The first phase starts immediately after the formalin injection and lasts about 10 min. This phase represents an acute form of pain response deriving from the direct activation of nociceptors. Conversely, the second phase that starts after 10 min from the formalin injection, represents a tonic form of pain deriving from the establishment of an inflammatory response sustained by mechanisms of central sensitization in the dorsal horn of the spinal cord (Coderre and Melzack, 1992; Tjølsen et al., 1992). As reported in **Figure 1**, there was no statistical difference in the formalin test between vehicle treated male and female mice (**Figures 1A,B**). However, while repeated fluoxetine administration did not affect formalin behavior in male mice (**Figure 1A**) in both phases, the second phase of the formalin test was significantly reduced in female mice (**Figure 1B**), suggesting a sex-dependent analgesic effect of chronic fluoxetine.

### Chronic Treatment with Fluoxetine Reduces the Expression of HDAC2 in DRG of Female Mice

**FIGURE 2:**
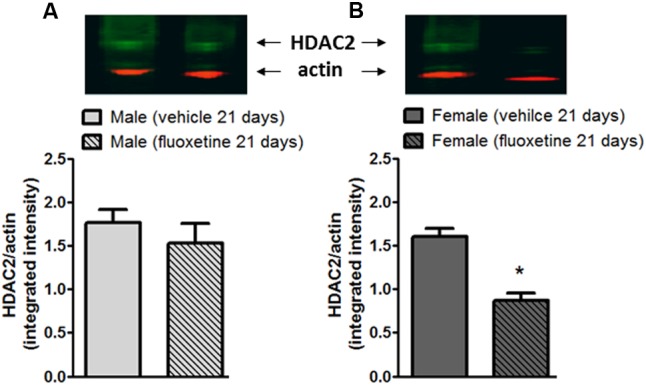
Expression of HDAC2 in the L4-L5 DRGs of male and female mice after chronic administration with fluoxetine. Chronic administration with fluoxetine (10 mg/Kg, i.p. for 21 days) selectively decreased the expression of HDAC2 in DRGs from female mice **(B)** but not in male mice **(A)**. (Upper panels) Representative western blot analysis for HDAC2 in DRGs. (Lower panels) Densitometric analysis of HDAC2 bands normalized by actin. Data are the means ± SEM of five animals. ^∗^*p* < 0.05 (Student’s *t*-test) versus values obtained in animals of the same sex treated with vehicle.

Fluoxetine is known to induce direct epigenetic modification including increased levels of acetylated histone H3 and H4 proteins (Vierci et al., 2016). Moreover, HDAC2 levels have been shown to be reduced after chronic fluoxetine in the brain (Faillace et al., 2015). Thus, we investigated whether HDAC2 levels are modified in the DRG after a chronic treatment with fluoxetine in male and female mice. After a 21-day treatment with fluoxetine (10 mg/kg, i.p.), L4–L6 DRGs were removed and processed for protein analysis. As reported in **Figure 2**, western blot analysis shows that levels of HDAC2 were significantly decreased only in female mice (**Figure 2B**) whereas no changes were observed in male mice (**Figure 2A**) indicating that chronic fluoxetine is able to reduce the expression of HDAC2 in a sex-specific manner.

### Effects of Chronic Treatment with Fluoxetine on HDAC2 and mGlu2 Receptor Expression in the Spinal DH of Male and Female Mice

By removing acetyl groups from histone proteins, HDACs promote an inactive state of chromatin resulting in the silencing of downstream genes. In particular, HDAC2 has been associated with a suppression on GRM2 gene encoding for the mGlu2 receptor (Kurita et al., 2012).

We combined immunohistochemistry and western blot analyses in order to evaluate whether HDAC2 expression is affected in the spinal dorsal horn of male and female mice after a 21-day treatment with fluoxetine (10 mg/kg, i.p.). Also, since the expression of mGlu2 receptor has been reported to be under the control of HDAC2 (Kurita et al., 2012), we tested whether mGlu2 receptor expression is affected by chronic treatment with fluoxetine.

**FIGURE 3:**
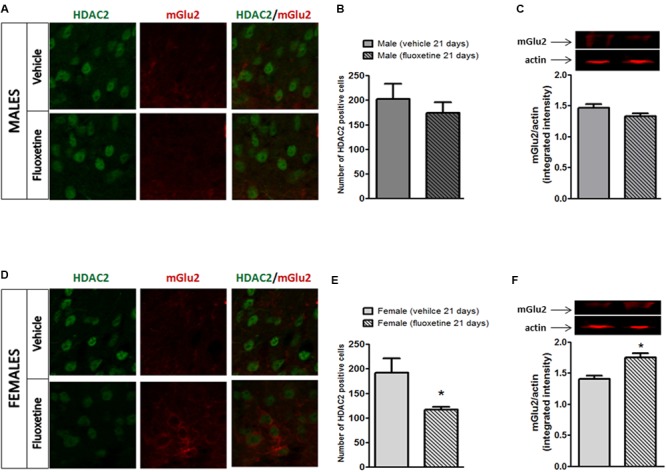
Expression of HDAC2 and mGlu2 receptors in the lumbar dorsal horn of male and female mice after chronic administration with fluoxetine. **(A,D)** Representative examples of HADC2 positive cells (left column) and mGlu2 immunostaining (central column) or overlapping (right column) in the dorsal horn of the spinal cord in male and female mice after chronic treatment with fluoxetine. **(B,E)** Quantification of the cell density (cell number/area) for HDAC2 immunopositive cells. Bars represent average ± SEM. ^∗^*p* < 0.05 (Student’s *t*-test) versus vehicle. **(C,F)** Densitometric analysis of mGlu2 bands normalized by actin. Data are the means ± SEM of five animals. ^∗^*p* < 0.05 (Student’s *t*-test) versus values obtained in animals of the same sex treated with vehicle.

As reported in **Figure 3**, no changes in HDAC2 expression were observed in male mice treated with fluoxetine (**Figures 3A,B**). Also no changes were detected in mGlu2 receptor expression in male mice (**Figure 3C**). Strikingly, a decrease in HDAC2 expression together with an increase in the expression of mGlu2 receptors was observed in female mice chronically treated with fluoxetine compared to vehicle treated female mice (**Figures 3A–C**).

### Effects of Chronic Treatment with Fluoxetine on Acetyl-Histone 3 Levels in the Spinal DH of Male and Female Mice

**FIGURE 4:**
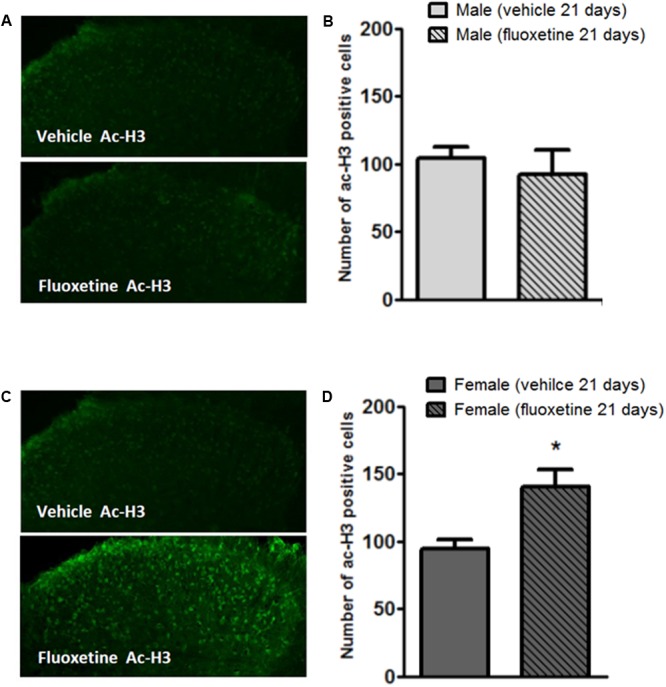
Modulation of acetyl-H3 positive cell density in the dorsal horn of male and female mice by chronic treatment with fluoxetine. **(A,C)** Representative examples of acetyl-H3 immunopositive cells in the mouse dorsal horn from male and female mice treated with vehicle or fluoxetine (10 mg/kg, i.p.) for 21 days. **(B,D)** Quantification of immunopositive cells. Bars represent average ± SEM of three animals. ^∗^*p* < 0.01 (Student’s *t*-test) versus values obtained in animals of the same sex treated with vehicle.

Since HDACs remove acetyl groups from histone proteins, we investigated whether the downregulation of HDAC2 affected levels of acetyl-H3 in the dorsal horn of the spinal cord. As expected, immunohistochemisty analysis in the spinal DH shows that chronic fluoxetine increases levels of acetyl-H3 specifically in female mice (**Figures 4C,D**) whereas no changes were observed in male mice (**Figures 4A,B**). This result is consistent with the reduced expression of HDAC2 in female mice (**Figures 3D,E**).

## Discussion

Several studies have shown gender differences in antidepressant efficacy (Kokras and Dalla, 2017) and a number of clinical studies have reported that depressed women respond to SSRIs therapy better than males (Haykal and Akiskal, 1999; Kornstein et al., 2000; Quitkin et al., 2002; Berlanga and Flores-Ramos, 2006). Although hormonal and pharmacokintec differences have been postulated as likely candidates to explain different responses to antidepressant pharmacotherapy between sex, the underlying mechanisms are still unknown (Sramek et al., 2016).

Differences in pain sensitivity are also seen in women that usually report greater pain than men (Unruh, 1996; Berkley, 1997; Riley et al., 1998; Fillingim et al., 2009) and antidepressants are effective in relieving chronic pain (Gilron et al., 2013). However, little is known about sex differences in response to chronic antidepressant treatment for the management of chronic pain. Moreover, although it is believed that depression can exacerbate pain, there is evidence that the antidepressant and the analgesic effects occur independently. Antidepressants are analgesic in patients with chronic pain and no concomitant depression (Saarto and Wiffen, 2005, 2007). In addition, the delay of action of antidepressants in chronic pain management in some studies is shorter than in depression (Arnold et al., 2005). This work shows for the first time that chronic administration of fluoxetine induces sex-dependend analgesic effects in mice. We also observed sex-specific changes in mGlu2 expression in the DH and DRGs of female mice together with a decreased expression of HDAC2.

Metabotropic glutamate receptors are known to modulate pain sensitivity (Chiechio and Nicoletti, 2012; Palazzo et al., 2014; Chiechio, 2016). In particular analgesia can be obtained by either blocking group I mGlu receptors, namely mGlu1 and mGlu5 (Walker et al., 2001; Sasikumar et al., 2010; Bennett et al., 2012; Brumfield et al., 2012) or by activating group II and group III mGlu receptors (Chiechio and Nicoletti, 2012; Chiechio, 2016; Johnson et al., 2017). Also, we have demonstrated that epigenetic drugs that increase the expression of mGlu2 receptors are analgesic in models of persistent pain (Chiechio et al., 2002, 2009, 2010; Zammataro et al., 2014).

Antidepressant drugs are known to induce analgesic effects. Different mechanisms have been demonstrated to explain their efficacy in reducing pain. However, long term regulation of gene expression and epigenetic mechanisms are likely to occur after chronic administration. In this regard, it is important to mention that different classes of antidepressant drugs have been demonstrated to have a plethora of effects in CNS disorders and are also known to have a role in neurogenesis (Wang et al., 2011; Masuda et al., 2012; Meneghini et al., 2014; Zhou et al., 2016; King et al., 2017). Many of these effects are sex-dependent (Gray and Hughes, 2015; Kokras and Dalla, 2017) and fluoxetine itself is known to induce sex specific effects on neurogenesis (Hodes et al., 2010). However, so far, no studies have investigated whether long-term treatment with antidepressant drugs affect pain behavior in a sex-dependent manner.

With this study we have shown that chronically administered fluoxetine induces sex-specific analgesic effects in the mouse formalin test and modulates the expression of the epigenetic modifying enzymes, HDAC2, and the expression of mGlu2 receptors in the spinal dorsal horn and DRG of female mice.

A number of HDAC inhibitors have been tested in different pain models and it has been shown that they have analgesic effects through different mechanisms (Chiechio et al., 2009, 2010; Bai et al., 2010; Zhang et al., 2011; Denk et al., 2013; Matsushita et al., 2013; Onofrj et al., 2013; Ximenes et al., 2013; Chen et al., 2014; Kukkar et al., 2014; Zammataro et al., 2014; Capasso et al., 2015; Cao et al., 2016; Tao et al., 2016; Alqinyah et al., 2017; Fork et al., 2017; Lin et al., 2017). We first demonstrated that HDAC inhibitors have analgesic properties via the upregulation of the metabotropic glutamate receptor type-2 (Chiechio et al., 2009, 2010; Onofrj et al., 2013), an inhibitory receptor whose activation or upregulation mediates analgesia (Chiechio et al., 2002, 2009, 2010; Onofrj et al., 2013; Zammataro et al., 2014; Chiechio, 2016). More recently, the isoform HDAC2 has been involved both in pain modulation (Kim et al., 2011; Zhao et al., 2011; Yang et al., 2015; Maiarù et al., 2016) and in the regulation of mGlu2 promoter activity (Kurita et al., 2012) and fluoxetine itself has been reported to regulate the expression of the HDAC2 isoform (Faillace et al., 2015).

In our experimental protocol we did not find sex differences in the formalin test in saline treated mice. Previous studies have reported sex differences in the mouse formalin test only in a late phase, phase 3, developing after 1 h of formalin injection, but not in phases 1 and 2 as observed in the present study (Kim et al., 1999). However, in pain studies phase 3 is rarely measured as it is considered less reproducible and relevant than phases 1 and 2 that reflect acute and tonic phase of pain, respectively (Coderre and Melzack, 1992; Tjølsen et al., 1992). Consistent with our findings, the lack of analgesic effect of chronically injected fluoxetine in male mice has been previously reported in a persistent pain model such as the sciatic nerve cuffing in mice, where, similarly to clinical observations, TCAs such as amitriptyline and nortriptyline were able to elicit analgesic effects while fluoxetine resulted ineffective (Benbouzid et al., 2008). Hormonal and genetic factors might influence pain sensitivity and the response to specific pharmacological treatments. Also, pharmacokinetic differences may account for the analgesic effects of fluoxetine in female mice. Female mice are known to metabolize fluoxetine and to produce more norfluoxetine than males in the brain (Hodes et al., 2010). On the other hand norfluoxetine has been shown to interact with estrogen receptors to regulate ER-mediated gene expression (Lupu et al., 2015). Furthermore, estradiol is able to reduce the expression of HDAC2 in the hippocampus (Zhao et al., 2010).

## Conclusion

In summary, this study provides evidence that antidepressant drugs might have sex-specific analgesic effects after chronic administration. The observation that fluoxetine induces sex specific changes in HDAC2 and mGlu2 expression in the dorsal horn of the spinal cord and in DRGs provides a molecular explanation for the analgesic effects in females and suggests that estrogens and/or estrogen receptors might play a role in the observed effects. Studies in ovariectomized female mice and estrogen replacement protocols are in progress to investigate mechanisms underlying the observed differences in male and female mice in response to a chronic treatment with fluoxetine. So far we hypothesize that fluoxetine induces analgesic effects in female mice by reducing the expression of HDAC2 and consequently upregulating the mGlu2 receptor, known to induce analgesia.

## Materials and Methods

### Animals

Male and female CD1 mice littermates aged between 8 and 12 weeks were used for these experiments. All experiments were done in accordance with experimental protocols approved by the animal study committee at University of Catania, Italy (IACUC project 161/2012).

### Drugs

The dose of fluoxetine was chosen according to previous studies (Singh et al., 2001; Iyengar et al., 2004; Jones et al., 2005; Zhao et al., 2007). In addition, we performed preliminary studies to identify a dose that produced analgesic effects without causing toxicity or motor impairment. To evaluate the effects of chronic administration of fluoxetine (10 mg/kg; Tocris) on acute and persistent pain, mice were randomly assigned to different groups by an independent researcher and intraperitoneally injected with vehicle (saline, 1 ml/kg) or fluoxetine (10 mg/kg) for 21 consecutive days.

### Behavioral Experiments

Littermate male and female mice aged between 8 and 9 weeks were acclimated to the experimental room and were used for behavioral tests by observers blind to the sex and the treatment of the animals.

### Formalin Test

Formalin (5%, 10 μl; Sigma–Aldrich) was injected subcutaneously into the plantar surface of the right hind paw, and the total time spent licking and flinching the injected paw was monitored for 1 h and recorded every 5 min as reported previously (Chiechio et al., 2009). Formalin scores were separated into two phases, phase I (0–10 min) and phase II (15–50 min). A mean response was then calculated for each phase. All tests were performed and analyzed blind to treatment.

### Immunohystochemical Staining

After the behavioral test mice were euthanized by CO_2_ inhalation and spinal cord was removed and kept in 4% paraformaldehyde at 4°C overnight and then in 30% sucrose for cryoprotection. Sections (20 μm thick) from the lumbar segment were sequentially collected using a sliding microtome and stored at -20°C in an anti freezer solution containing 30% ethylene glycol in 0.1 M phosphate buffer (PBS). For immunohystochemical staining, spinal cord sections from the lumbar segment were sequentially incubated with rabbit anti-HDAC2 (1:200, Abcam) and mouse anti-mGlu2 (1:250 Abcam) in PBS containing 3% bovine serum albumin (BSA, Sigma) and 0.1% Triton X-100 overnight. The following fluorescent secondary antibodies were used: donkey anti-rabbit (1:200; Jackson Immuno Research) for 2 h, and Cy3-conjugated streptavidin (1:1000; Jackson Immuno Research) for 1 h. After washing with PBS, sections were coverslipped with a mounting medium for fluorescence (Sigma). Fluorescent signal was detected using a Zeiss Axio Observer.Z1 microscope equipped with the Apotome.2 acquisition system (Carl Zeiss S.p.A, Milan, Italy).

Quantification and colocalization of positive cells in the dorsal horn were performed by an investigator blind to the treatment using Image-J software. At least three fields per slices from five slices per animals were analyzed. Three different mice/group were used. Statistical analysis was performed by using Student’s *t*-test comparing treatment versus vehicle in animals of the same sex.

### Western Blot

Mice were euthanized by CO_2_ inhalation and L5–L6 dorsal root ganglia and the lumbar segment of the spinal cord of male and female CD1 mice were removed and homogenized. Ten micrograms of total protein were separated by 10% SDS-PAGE and electrophoretically transferred onto protein-sensitive PVDF transfer membranes (Thermo Scientific). Membranes were blocked in Odyssey blocker (LI-COR) for 1 h at room temperature and rabbit anti-HDAC2 (1:1000, Abcam), mouse anti-mGlu2 (1:1000, Abcam) or mouse anti-actin (1:1000, Sigma–Aldrich) primary antibody were simultaneously used for immunoblotting overnight at 4°C. A goat anti-rabbit antibody labeled with IRD800CW and a goat anti-mouse antibody labeled with Alexa 680 (LICOR) were incubated for 1 h at room temperature. Proteins were detected with the Odyssey Infrared Fluorescence Imaging System (LI-COR) and protein quantification was expressed as integrated intensity.

### Statistical Analysis

Sample size was calculated with the GPower software. *A priori* analysis was performed by *F*-test (ANOVA) with the following input parameters: alpha = 0.05, power 1-beta = 0.95, *F* = 0,40 resulting in a total sample size of 56 mice. Behavioral data are presented as mean ± SEM of at least nine animals per group. Statistical comparisons were performed with Student’s *t*-test using the statistical software package GraphPad Prism. Western blot and immunochemical analyses are presented as mean ± SEM of at least three independent experiments performed in triplicate. Statistical significance was determined using unpaired *t*-tests. In all cases, *p* < 0.05 was considered statistically significant.

## Ethics Statement

All experiments were approved by the local animal ethics committee.

## Author Contributions

The study was conceived by MZ and SC. Behavioral experiments were designed by MZ, HH, CP, and SC. Immunohistochemistry and western blot experiments were designed by SM, MB, MS, and SC. All authors contributed to writing the manuscript, and all read and approved the final manuscript.

## Conflict of Interest Statement

The authors declare that the research was conducted in the absence of any commercial or financial relationships that could be construed as a potential conflict of interest.

## References

[B1] Alqinyah M. Maganti N. Ali M. W. Yadav R. Gao M. Cacan E. (2017). Regulator of G protein signaling 10 (Rgs10) expression is transcriptionally silenced in activated microglia by histone deacetylase activity. *Mol. Pharmacol.* 91 197–207. 10.1124/mol.116.106963 28031332 PMC5325084

[B2] Arnold L. M. Hess E. V. Hudson J. I. Welge J. A. Berno S. E. Keck P. E. Jr. (2002). A randomized, placebo-controlled, double-blind, flexible-dose study of fluoxetine in the treatment of women with fibromyalgia. *Am. J. Med.* 112 191–197. 10.1016/S0002-9343(01)01089-0 11893345

[B3] Arnold L. M. Rosen A. Pritchett Y. L. D’Souza D. N. Goldstein D. J. Iyengar S. (2005). A randomized, double-blind, placebo-controlled trial of duloxetine in the treatment of women with fibromyalgia with or without major depressive disorder. *Pain* 119 5–15. 10.1016/j.pain.2005.06.031 16298061

[B4] Attal N. Cruccu G. Haanpää M. Hansson P. Jensen T. S. Nurmikko T. (2006). EFNS Task Force. EFNS guidelines on pharmacological treatment of neuropathic pain. *Eur. J. Neurol.* 13 1153–1169. 10.1111/j.1468-1331.2006.01511.x 17038030

[B5] Bai G. Wei D. Zou S. Ren K. Dubner R. (2010). Inhibition of class II histone deacetylases in the spinal cord attenuates inflammatory hyperalgesia. *Mol. Pain* 6:51. 10.1186/1744-8069-6-51 20822541 PMC2942827

[B6] Benbouzid M. Choucair-Jaafar N. Yalcin I. Waltisperger E. Muller A. Freund-Mercier M. J. (2008). Chronic, but not acute, tricyclic antidepressant treatment alleviates neuropathic allodynia after sciatic nerve cuffing in mice. *Eur. J. Pain* 12 1008–1017. 10.1016/j.ejpain.2008.01.010 18331804

[B7] Bennett C. E. Burnett D. A. Greenlee W. J. Knutson C. E. Korakas P. Li C. (2012). Fused tricyclic mGluR1 antagonists for the treatment of neuropathic pain. *Bioorg. Med. Chem. Lett.* 22 1575–1578. 10.1016/j.bmcl.2011.12.131 22266036

[B8] Berkley K. J. (1997). Sex differences in pain. *Behav. Brain Sci.* 20 371–380. 10.1017/S0140525X97221485 10097000

[B9] Berlanga C. Flores-Ramos M. (2006). Different gender response to serotonergic and noradrenergic antidepressants. A comparative study of the efficacy of citalopram and reboxetine. *J. Affect. Disord.* 95 119–123. 10.1016/j.jad.2006.04.029 16782204

[B10] Berton O. Nestler E. J. (2006). New approaches to antidepressant drug discovery: beyond monoamines. *Nat. Rev. Neurosci.* 7 137–151. 10.1038/nrn1846 16429123

[B11] Bomholt S. F. Mikkelsen J. D. Blackburn-Munro G. (2005). Antinociceptive effects of the antidepressants amitriptyline, duloxetine, mirtazapine and citalopram in animal models of acute, persistent and neuropathic pain. *Neuropharmacology* 48 252–263. 10.1016/j.neuropharm.2004.09.012 15695164

[B12] Borrelli E. Nestler E. J. Allis C. D. Sassone-Corsi P. (2008). Decoding the epigenetic language of neuronal plasticity. *Neuron* 60 961–974. 10.1016/j.neuron.2008.10.012 19109904 PMC2737473

[B13] Brumfield S. Korakas P. Silverman L. S. Tulshian D. Matasi J. J. Qiang L. (2012). Synthesis and SAR development of novel mGluR1 antagonists for the treatment of chronic pain. *Bioorg. Med. Chem. Lett.* 22 7223–7226. 10.1016/j.bmcl.2012.09.048 23084894

[B14] Cantini F. Bellandi F. Niccoli L. Di Munno O. (1994). Fluoxetin combined with cyclobenzaprine in the treatment of fibromyalgia. *Minerva Med.* 85 97–100. 8196850

[B15] Cao D. Y. Bai G. Ji Y. Karpowicz J. M. Traub R. J. (2016). EXPRESS: histone hyperacetylation modulates spinal type II metabotropic glutamate receptor alleviating stress-induced visceral hypersensitivity in female rats. *Mol. Pain* 12:1744806916660722. 10.1177/1744806916660722 27385724 PMC4956148

[B16] Capasso K. E. Manners M. T. Quershi R. A. Tian Y. Gao R. Hu H. (2015). Effect of histone deacetylase inhibitor JNJ-26481585 in pain. *J. Mol. Neurosci.* 55 570–578. 10.1007/s12031-014-0391-7 25085711

[B17] Chen H. Y. Li L. Fu Z. J. (2014). Histone deacetylase inhibitors trichostatin A and suberoylanilide hydroxamic acid attenuate ventilator-induced lung injury. *Pharmazie* 69 55–59. 24601225

[B18] Chiechio S. (2016). Modulation of chronic pain by metabotropic glutamate receptors. *Adv. Pharmacol.* 75 63–89. 10.1016/bs.apha.2015.11.001 26920009

[B19] Chiechio S. Caricasole A. Barletta E. Storto M. Catania M. V. Copani A. (2002). L-Acetylcarnitine induces analgesia by selectively up-regulating mGlu2 metabotropic glutamate receptors. *Mol. Pharmacol.* 61 989–996. 10.1124/mol.61.5.989 11961116

[B20] Chiechio S. Copani A. Zammataro M. Battaglia G. Gereau R. W. IV Nicoletti F. (2010). Transcriptional regulation of type-2 metabotropic glutamate receptors: an epigenetic path to novel treatments for chronic pain. *Trends Pharmacol. Sci.* 31 153–160. 10.1016/j.tips.2009.12.003 20064669

[B21] Chiechio S. Nicoletti F. (2012). Metabotropic glutamate receptors, and the control of chronic pain. *Curr. Opin. Pharmacol.* 12 28–34. 10.1016/j.coph.2011.10.010 22040745

[B22] Chiechio S. Zammataro M. Morales M. E. Busceti C. L. Drago F. Gereau R. W. IV. (2009). Epigenetic modulation of mGlu2 receptors by histone deacetylase inhibitors in the treatment of inflammatory pain. *Mol. Pharmacol.* 75 1014–1020. 10.1124/mol.108.054346 19255242

[B23] Coderre T. J. Melzack R. (1992). The contribution of excitatory amino acids to central sensitization and persistent nociception after formalin-induced tissue injury. *J. Neurosci.* 12 3665–3670. 1326610 10.1523/JNEUROSCI.12-09-03665.1992 PMC6575737

[B24] Craft R. M. (2003). Sex differences in opioid analgesia: “from mouse to man”. *Clin. J. Pain* 19 175–186. 10.1097/00002508-200305000-00005 12792556

[B25] Denk F. Huang W. Sidders B. Bithell A. Crow M. Grist J. (2013). HDAC inhibitors attenuate the development of hypersensitivity in models of neuropathic pain. *Pain* 154 1668–1679. 10.1016/j.pain.2013.05.021 23693161 PMC3763368

[B26] Dharmshaktu P. Tayal V. Kalra B. S. (2012). Efficacy of antidepressants as analgesics: a review. *J. Clin. Pharmacol.* 52 6–17. 10.1177/0091270010394852 21415285

[B27] Erburu M. Muñoz-Cobo I. Domínguez-Andrés J. Beltran E. Suzuki T. Mai A. (2015). Chronic stress and antidepressant induced changes in Hdac5 and Sirt2 affect synaptic plasticity. *Eur. Neuropsychopharmacol.* 25 2036–2048. 10.1016/j.euroneuro.2015.08.016 26433268

[B28] Faillace M. P. Zwiller J. Bernabeu R. O. (2015). Effects of combined nicotine and fluoxetine treatment on adult hippocampal neurogenesis and conditioned place preference. *Neuroscience* 300 104–115. 10.1016/j.neuroscience.2015.05.017 25981209

[B29] Fillingim R. B. King C. D. Ribeiro-Dasilva M. C. Rahim-Williams B. Riley J. L. III. (2009). Sex, gender, and pain: a review of recent clinical and experimental findings. *J. Pain* 10 447–485. 10.1016/j.jpain.2008.12.001 19411059 PMC2677686

[B30] Finnerup N. B. Attal N. Haroutounian S. McNicol E. Baron R. Dworkin R. H. (2015). Pharmacotherapy for neuropathic pain in adults: a systematic review and meta-analysis. *Lancet Neurol.* 14 162–173. 10.1016/S1474-4422(14)70251-0 25575710 PMC4493167

[B31] Finnerup N. B. Sindrup S. H. Jensen T. S. (2010). The evidence for pharmacological treatment of neuropathic pain. *Pain* 150 573–581. 10.1016/j.pain.2010.06.019 20705215

[B32] Fork C. Vasconez A. E. Janetzko P. Angioni C. Schreiber Y. Ferreirós N. (2017). Epigenetic control of microsomal prostaglandin E synthase-1 by HDAC-mediated recruitment of p300. *J. Lipid Res.* 58 386–392. 10.1194/jlr.M072280 27913583 PMC5282954

[B33] Gilron I. Jensen T. S. Dickenson A. H. (2013). Combination pharmacotherapy for management of chronic pain: from bench to bedside. *Lancet Neurol.* 12 1084–1095. 10.1016/S1474-4422(13)70193-5 24074723

[B34] Goldenberg D. Mayskiy M. Mossey C. Ruthazer R. Schmid C. (1996). A randomized, double-blind crossover trial of fluoxetine and amitriptyline in the treatment of fibromyalgia. *Arthritis Rheum.* 39 1852–1859. 10.1002/art.1780391111 8912507

[B35] Gray V. C. Hughes R. N. (2015). Drug-, dose- and sex-dependent effects of chronic fluoxetine, reboxetine and venlafaxine on open-field behavior and spatial memory in rats. *Behav. Brain Res.* 281 43–54. 10.1016/j.bbr.2014.12.023 25523028

[B36] Grozinger C. M. Schreiber S. L. (2002). Deacetylase enzymes: biological functions and the use of small-molecule inhibitors. *Chem. Biol.* 9 3–16. 10.1016/S1074-5521(02)00092-3 11841934

[B37] Haykal R. F. Akiskal H. S. (1999). The long-term outcome of dysthymia in private practice: clinical features, temperament, and the art of management. *J. Clin. Psychiatry* 60 508–518. 10.4088/JCP.v60n0802 10485632

[B38] Hodes G. E. Hill-Smith T. E. Suckow R. F. Cooper T. B. Lucki I. (2010). Sex-specific effects of chronic fluoxetine treatment on neuroplasticity and pharmacokinetics in mice. *J. Pharmacol. Exp. Ther.* 332 266–273. 10.1124/jpet.109.158717 19828877 PMC2802485

[B39] Iyengar S. Webster A. A. Hemrick-Luecke S. K. Xu J. Y. Simmons R. M. (2004). Efficacy of duloxetine, a potent and balanced serotonin-norepinephrine reuptake inhibitor in persistent pain models in rats. *J. Pharmacol. Exp. Ther.* 311 576–584. 10.1124/jpet.104.070656 15254142

[B40] Johnson M. P. Muhlhauser M. A. Nisenbaum E. S. Simmons R. M. Forster B. M. Knopp K. L. (2017). Broad spectrum efficacy with LY2969822, an oral prodrug of metabotropic glutamate 2/3 receptor agonist LY2934747, in rodent pain models. *Br. J. Pharmacol.* 174 822–835. 10.1111/bph.13740 28177520 PMC5386998

[B41] Jones C. K. Peters S. C. Shannon H. E. (2005). Efficacy of duloxetine, a potent and balanced serotonergic and noradrenergic reuptake inhibitor, in inflammatory and acute pain models in rodents. *J. Pharmacol. Exp. Ther.* 312 726–732. 10.1124/jpet.104.075960 15494550

[B42] Kim D. K. Hwang C. K. Wagley Y. Law P. Y. Wei L. N. Loh H. H. (2011). p38 mitogen-activated protein kinase and PI3-kinase are involved in up-regulation of mu opioid receptor transcription induced by cycloheximide. *J. Neurochem.* 116 1077–1087. 10.1111/j.1471-4159.2010.07163.x 21198637 PMC3078638

[B43] Kim S. J. Calejesan A. A. Li P. Wei F. Zhuo M. (1999). Sex differences in late behavioral response to subcutaneous formalin injection in mice. *Brain Res.* 829 185–189. 10.1016/S0006-8993(99)01353-0 10350546

[B44] King J. R. Velasquez J. C. Torii M. Bonnin A. (2017). Effect of maternal ± citalopram exposure on P11 expression and neurogenesis in the mouse fetal brain. *ACS Chem. Neurosci.* 8 1019–1025. 10.1021/acschemneuro.6b00339 28076682 PMC5453513

[B45] Kokras N. Dalla C. (2017). Preclinical sex differences in depression and antidepressant response: implications for clinical research. *J. Neurosci. Res.* 95 731–736. 10.1002/jnr.23861 27870451

[B46] Kornstein S. G. Schatzberg A. F. Thase M. E. Yonkers K. A. McCullough J. P. Keitner G. I. (2000). Gender differences in treatment response to sertraline versus imipramine in chronic depression. *Am. J. Psychiatry* 157 1445–1452. 10.1176/appi.ajp.157.9.1445 10964861

[B47] Kukkar A. Singh N. Jaggi A. S. (2014). Attenuation of neuropathic pain by sodium butyrate in an experimental model of chronic constriction injury in rats. *J. Formos. Med. Assoc.* 113 921–928. 10.1016/j.jfma.2013.05.013 23870713

[B48] Kurita M. Holloway T. García-Bea A. Kozlenkov A. Friedman A. K. Moreno J. L. (2012). HDAC2 regulates atypical antipsychotic responses through the modulation of mGlu2 promoter activity. *Nat. Neurosci.* 15 1245–1254. 10.1038/nn.3181 22864611 PMC3431440

[B49] Lin C. R. Cheng J. K. Wu C. H. Chen K. H. Liu C. K. (2017). Epigenetic suppression of potassium-chloride co-transporter 2 expression in inflammatory pain induced by complete Freund’s adjuvant (CFA). *Eur. J. Pain* 21 309–321. 10.1002/ejp.925 27506893

[B50] Lupu D. Pop A. Cherfan J. Kiss B. Loghin F. (2015). In vitro modulation of estrogen receptor activity by norfluoxetine. *Clujul Med.* 88 386–390. 10.15386/cjmed-476 26609274 PMC4632900

[B51] Mai A. Massa S. Rotili D. Cerbara I. Valente S. Pezzi R. (2005). Histone deacetylation in epigenetics: an attractive target for anticancer therapy. *Med. Res. Rev.* 25 261–309. 10.1002/med.20024 15717297

[B52] Maiarù M. Morgan O. B. Tochiki K. K. Hobbiger E. J. Rajani K. Overington D. W. (2016). Complex regulation of the regulator of synaptic plasticity histone deacetylase 2 in the rodent dorsal horn after peripheral injury. *J. Neurochem.* 138 222–232. 10.1111/jnc.13621 26998823 PMC4982040

[B53] Masuda T. Nakagawa S. Boku S. Nishikawa H. Takamura N. Kato A. (2012). Noradrenaline increases neural precursor cells derived from adult rat dentate gyrus through β2 receptor. *Prog. Neuropsychopharmacol. Biol. Psychiatry.* 36 44–51. 10.1016/j.pnpbp.2011.08.019 21914456

[B54] Matsushita Y. Araki K. Omotuyi O. I. Mukae T. Ueda H. (2013). HDAC inhibitors restore C-fibre sensitivity in experimental neuropathic pain model. *Br. J. Pharmacol.* 170 991–998. 10.1111/bph.12366 24032674 PMC3949648

[B55] Max M. B. Lynch S. A. Muir J. Shoaf S. E. Smoller B. Dubner R. (1992). Effects of desipramine, amitriptyline, and fluoxetine on pain in diabetic neuropathy. *N. Engl. J. Med.* 326 1250–1256. 10.1056/NEJM199205073261904 1560801

[B56] Meneghini V. Cuccurazzu B. Bortolotto V. Ramazzotti V. Ubezio F. Tzschentke T. M. (2014). The noradrenergic component in tapentadol action counteracts μ-opioid receptor-mediated adverse effects on adult neurogenesis. *Mol. Pharmacol.* 85 658–670. 10.1124/mol.113.091520 24516101

[B57] Menke A. Binder E. B. (2014). Epigenetic alterations in depression and antidepressant treatment. *Dialogues Clin. Neurosci.* 16 395–404. 25364288 10.31887/DCNS.2014.16.3/amenke PMC4214180

[B58] Menke A. Klengel T. Binder E. B. (2012). Epigenetics, depression and antidepressant treatment. *Curr. Pharm. Des.* 18 5879–5889. 10.2174/138161212803523590 22681167

[B59] Mika J. Zychowska M. Makuch W. Rojewska E. Przewlocka B. (2013). Neuronal and immunological basis of action of antidepressants in chronic pain - clinical and experimental studies. *Pharmacol. Rep.* 65 1611–1621. 10.1016/S1734-1140(13)71522-6 24553009

[B60] Millan M. J. (2002). Descending control of pain. *Prog. Neurobiol.* 66 355–474. 10.1016/S0301-0082(02)00009-6 12034378

[B61] Mogil J. S. Wilson S. G. Chesler E. J. Rankin A. L. Nemmani K. V. Lariviere W. R. (2003). The melanocortin-1 receptor gene mediates female-specific mechanisms of analgesia in mice and humans. *Proc. Natl. Acad. Sci. U.S.A.* 100 4867–4872. 10.1073/pnas.0730053100 12663858 PMC153647

[B62] Newell-Price J. Clark A. J. King P. (2000). DNA methylation and silencing of gene expression. *Trends Endocrinol. Metab.* 11 142–148. 10.1016/S1043-2760(00)00248-4 10754536

[B63] Obata H. Conklin D. Eisenach J. C. (2005). Spinal noradrenaline transporter inhibition by reboxetine and Xen2174 reduces tactile hypersensitivity after surgery in rats. *Pain* 113 271–276. 10.1016/j.pain.2004.10.017 15661433

[B64] Onofrj M. Ciccocioppo F. Varanese S. di Muzio A. Calvani M. Chiechio S. (2013). Acetyl-L-carnitine: from a biological curiosity to a drug for the peripheral nervous system and beyond. *Expert Rev. Neurother.* 13 925–936. 10.1586/14737175.2013.814930 23965166

[B65] Palazzo E. Marabese I. de Novellis V. Rossi F. Maione S. (2014). Supraspinal metabotropic glutamate receptors: a target for pain relief and beyond. *Eur. J. Neurosci.* 39 444–454. 10.1111/ejn.12398 24494684

[B66] Perisic T. Zimmermann N. Kirmeier T. Asmus M. Tuorto F. Uhr M. (2010). Valproate and amitriptyline exert common and divergent influences on global and gene promoter-specific chromatin modifications in rat primary astrocytes. *Neuropsychopharmacology* 35 792–805. 10.1038/npp.2009 19924110 PMC3055607

[B67] Quitkin F. M. Stewart J. W. McGrath P. J. Taylor B. P. Tisminetzky M. S. Petkova E. (2002). Are there differences between women’s and men’s antidepressant responses? *Am. J. Psychiatry* 159 1848–1854. 10.1176/appi.ajp.159.11.1848 12411218

[B68] Riley J. L. III. Robinson M. E. Wise E. A. Myers C. D. Fillingim R. B. (1998). Sex differences in the perception of noxious experimental stimuli: a meta-analysis. *Pain* 74 181–187. 10.1016/S0304-3959(97)00199-1 9520232

[B69] Saarto T. Wiffen P. J. (2005). Antidepressants for neuropathic pain. *Cochrane Database Syst. Rev.* 20:CD005454. 10.1002/14651858.CD005454 16034979

[B70] Saarto T. Wiffen P. J. (2007). Antidepressants for neuropathic pain. *Cochrane Database Syst. Rev.* 17:CD005454. 10.1002/14651858.CD005454.pub2 17943857 PMC10576544

[B71] Sasikumar T. K. Qiang L. Burnett D. A. Greenlee W. J. Li C. Grilli M. (2010). A-ring modifications on the triazafluorenone core structure and their mGluR1 antagonist properties. *Bioorg. Med. Chem. Lett.* 20 2474–2477. 10.1016/j.bmcl.2010.03.004 20346665

[B72] Sharma N. L. Groselj B. Hamdy F. C. Kiltie A. E. (2013). The emerging role of histone deacetylase (HDAC) inhibitors in urological cancers. *BJU Int.* 111 537–542. 10.1111/j.1464-410X.2012.11647.x 23551441

[B73] Sindrup S. H. Bjerre U. Dejgaard A. Brøsen K. Aaes-Jørgensen T. Gram L. F. (1992). The selective serotonin reuptake inhibitor citalopram relieves the symptoms of diabetic neuropathy. *Clin. Pharmacol. Ther.* 52 547–552. 10.1038/clpt.1992.183 1424428

[B74] Sindrup S. H. Gram L. F. Brøsen K. Eshøj O. Mogensen E. F. (1990). The selective serotonin reuptake inhibitor paroxetine is effective in the treatment of diabetic neuropathy symptoms. *Pain* 42 135–144. 10.1016/0304-3959(90)91157-E 2147235

[B75] Sindrup S. H. Otto M. Finnerup N. B. Jensen T. S. (2005). Antidepressants in the treatment of neuropathic pain. *Basic Clin. Pharmacol. Toxicol.* 96 399–409. 10.1111/j.1742-7843.2005.pto_96696601.x 15910402

[B76] Singh V. P. Jain N. K. Kulkarni S. K. (2001). On the antinociceptive effect of fluoxetine, a selective serotonin reuptake inhibitor. *Brain Res.* 915 218–226. 10.1016/S0006-8993(01)02854-2 11595211

[B77] Sorge R. E. Mapplebeck J. C. Rosen S. Beggs S. Taves S. Alexander J. K. (2015). Different immune cells mediate mechanical pain hypersensitivity in male and female mice. *Nat. Neurosci.* 18 1081–1083. 10.1038/nn.4053 26120961 PMC4772157

[B78] Sramek J. J. Murphy M. F. Cutler N. R. (2016). Sex differences in the psychopharmacological treatment of depression. *Dialogues Clin. Neurosci.* 18 447–457. 28179816 10.31887/DCNS.2016.18.4/ncutler PMC5286730

[B79] Tao W. Zhou W. Wang Y. Sun T. Wang H. Zhang Z. (2016). Histone deacetylase inhibitor-induced emergence of synaptic δ-opioid receptors and behavioral antinociception in persistent neuropathic pain. *Neuroscience* 339 54–63. 10.1016/j.neuroscience.2016.09.015 27646288

[B80] Tjølsen A. Berge O. G. Hunskaar S. Rosland J. H. Hole K. (1992). The formalin test: an evaluation of the method. *Pain* 51 5–17. 10.1016/0304-3959(92)90003-T 1454405

[B81] Unruh A. M. (1996). Gender variations in clinical pain experience. *Pain* 65 123–167. 10.1016/0304-3959(95)00214-6 8826503

[B82] Vierci G. Pannunzio B. Bornia N. Rossi F. M. (2016). H3 and H4 lysine acetylation correlates with developmental and experimentally induced adult experience-dependent plasticity in the mouse visual cortex. *J. Exp. Neurosci.* 10 49–64. 10.4137/JEN.S39888 27891053 PMC5117113

[B83] Walker K. Bowes M. Panesar M. Davis A. Gentry C. Kesingland A. (2001). Metabotropic glutamate receptor subtype 5 (mGlu5) and nociceptive function. I. Selective blockade of mGlu5 receptors in models of acute, persistent and chronic pain. *Neuropharmacology* 40 1–9. 10.1016/S0028-3908(00)00113-1 11077065

[B84] Walker Z. Walker R. W. Robertson M. M. Stansfeld S. (1998). Antidepressant treatment of chronic tension-type headache: a comparison between fluoxetine and desipramine. *Headache* 38 523–528. 10.1046/j.1526-4610.1998.3807523.x 15613168

[B85] Wang Y. Neumann M. Hansen K. Hong S. M. Kim S. Noble-Haeusslein L. J. (2011). Fluoxetine increases hippocampal neurogenesis and induces epigenetic factors but does not improve functional recovery after traumatic brain injury. *J. Neurotrauma* 28 259–268. 10.1089/neu.2010.1648 21175261 PMC5206695

[B86] Wolfe F. Cathey M. A. Hawley D. J. (1994). A double-blind placebo controlled trial of fluoxetine in fibromyalgia. *Scand. J. Rheumatol.* 23 255–259. 10.3109/03009749409103725 7973479

[B87] Wolfe F. Ross K. Anderson J. Russell I. J. Hebert L. (1995). The prevalence and characteristics of fibromyalgia in the general population. *Arthritis Rheum.* 38 19–28. 10.1002/art.1780380104 7818567

[B88] Ximenes J. C. de Oliveira Gonçalves D. Siqueira R. M. Neves K. R. Santos Cerqueira G. Correia A. O. (2013). Valproic acid: an anticonvulsant drug with potent antinociceptive and anti-inflammatory properties. *Naunyn Schmiedebergs Arch. Pharmacol.* 386 575–587. 10.1007/s00210-013-0853-4 23584602

[B89] Yang F. Yang Y. Wang Y. Yang F. Li C. L. Wang X. L. (2015). Selective class I histone deacetylase inhibitors suppress persistent spontaneous nociception and thermal hypersensitivity in a rat model of bee venom-induced inflammatory pain. *Sheng Li Xue Bao* 67 447–454. 26490061

[B90] Zammataro M. Sortino M. A. Parenti C. Gereau R. W. IV. Chiechio S. (2014). HDAC and HAT inhibitors differently affect analgesia mediated by group II metabotropic glutamate receptors. *Mol. Pain* 10:68. 10.1186/1744-8069-10-68 25406541 PMC4247606

[B91] Zhang Z. Cai Y. Q. Zou F. Bie B. Pan Z. Z. (2011). Epigenetic suppression of GAD65 expression mediates persistent pain. *Nat. Med.* 17 1448–1455. 10.1038/nm.2442 21983856 PMC3210928

[B92] Zhao T. Liu X. Zhen X. Guo S. W. (2011). Levo-tetrahydropalmatine retards the growth of ectopic endometrial implants and alleviates generalized hyperalgesia in experimentally induced endometriosis in rats. *Reprod. Sci.* 18 28–45. 10.1177/1933719110381928 20884991

[B93] Zhao Z. Fan L. Frick K. M. (2010). Epigenetic alterations regulate estradiol-induced enhancement of memory consolidation. *Proc. Natl. Acad. Sci. U.S.A.* 107 5605–5610. 10.1073/pnas.0910578107 20212170 PMC2851775

[B94] Zhao Z. Q. Chiechio S. Sun Y. G. Zhang K. H. Zhao C. S. Scott M. (2007). Mice lacking central serotonergic neurons show enhanced inflammatory pain and an impaired analgesic response to antidepressant drugs. *J. Neurosci.* 27 6045–6053. 10.1523/JNEUROSCI.1623-07.2007 17537976 PMC6672267

[B95] Zhou Q. G. Lee D. Ro E. J. Suh H. (2016). Regional-specific effect of fluoxetine on rapidly dividing progenitors along the dorsoventral axis of the hippocampus. *Sci. Rep.* 6:35572. 10.1038/srep35572 27759049 PMC5069667

[B96] Zimmermann N. Zschocke J. Perisic T. Yu S. Holsboer F. Rein T. (2012). Antidepressants inhibit DNA methyltransferase 1 through reducing G9a levels. *Biochem. J.* 448 93–102. 10.1042/BJ20120674 22880885

